# Approach to Endoscopic Procedures: A Routine Protocol from a Quaternary University Referral Center Exclusively for Coronavirus Disease 2019 Patients

**DOI:** 10.6061/clinics/2020/e1989

**Published:** 2020-06-04

**Authors:** Tomazo Antonio Prince Franzini, Ana Paula Samy Tanaka Kotinda, Diogo Turiani Hourneaux de Moura, Márcia Lopes Vicente Badana, Marion Sielfeld de Medeiros, Patrícia Goulart Rodrigues Lima, Brigitte Feiner de Mello, Rafael Priante Kayano, Maria José Carvalho Carmona, Marcelo Cristiano Rocha, Aléia Faustina Campos, Thomas R. McCarty, Thaís Guimarães, Maria Luisa do Nascimento Moura, Christopher C. Thompson, Eduardo Guimarães Hourneaux de Moura

**Affiliations:** I Unidade de Endoscopia Gastrointestinal, Departamento de Gastroenterologia, Hospital das Clinicas HCFMUSP, Faculdade de Medicina, Universidade de Sao Paulo, Sao Paulo, SP, BR; II Division of Gastroenterology, Hepatology and Endoscopy, Brigham and Women’s Hospital, Boston, MA, USA; III Divisao de Molestias Infecciosas e Parasitarias, Hospital das Clinicas HCFMUSP, Faculdade de Medicina, Universidade de Sao Paulo, Sao Paulo, SP, BR; IV Divisao de Clinica Cirurgica III, Hospital das Clinicas HCFMUSP, Faculdade de Medicina, Universidade de Sao Paulo, Sao Paulo, SP, BR; V Divisao de Molestias Infecciosas e Parasitarias, Hospital das Clinicas HCFMUSP, Faculdade de Medicina, Universidade de Sao Paulo, Sao Paulo, SP, BR; VI Division of Gastroenterology, Hepatology and Endoscopy, Brigham and Women’s Hospital, Harvard Medical School, Boston, MA, USA

**Keywords:** COVID-19, Endoscopy, Coronavirus, SARS-CoV-2

## Abstract

**OBJECTIVES:**

The present coronavirus disease (COVID-19) pandemic has ushered in an unprecedented era of quality control that has necessitated advanced safety precautions and the need to ensure the adequate protection of healthcare professionals (HCPs). Endoscopy units, endoscopists, and other HCP may be at a significant risk for transmission of the virus. Given the immense burden on the healthcare system and surge in the number of patients with COVID-19, well-designed protocols and recommendations are needed. We aimed to systematically characterize our approach to endoscopic procedures in a quaternary university hospital setting and provide summary protocol recommendations.

**METHOD:**

This descriptive study details a COVID-19-specific protocol designed to minimize infection risks to patients and healthcare workers in the endoscopy unit.

**RESULTS:**

Our institution, located in São Paulo, Brazil, includes a 900-bed hospital, with a 200-bed-specific intensive care unit exclusively designed for patients with moderate and severe COVID-19. We highlighted recommendations for infection prevention and control during endoscopic procedures, including appropriate triage and screening, outpatient management and procedural recommendations, role and usage of personal protective equipment (PPE), and role and procedural logistics involving COVID-19-positive patients. We also detailed hospital protocols for reprocessing endoscopes and cleaning rooms and also provided recommendations to minimize severe acute respiratory syndrome coronavirus 2 transmission.

**CONCLUSION:**

This COVID-19-specific administrative and clinical protocol can be replicated or adapted in multiple institutions and endoscopy units worldwide. Furthermore, the recommendations and summary protocol may improve patient and HCP safety in these trying times.

## INTRODUCTION

In late November 2019, an outbreak of a viral respiratory illness in Wuhan, the capital city of Hubei province in China, attracted worldwide attention. This infection rapidly spread, and within a few weeks, it was declared as a public health emergency of international concern by the World Health Organization (WHO). This single-stranded RNA virus, later named severe acute respiratory syndrome coronavirus 2 (SARS-CoV-2) and identified to cause coronavirus disease (COVID-19), has become a global pandemic affecting over 211 countries to date (1). As of April 2020, over 2.7 million individuals have tested positive for SARS-CoV-2 infection, with more than 180,000 associated deaths worldwide (2). Given the immense burden on the healthcare system and surge in infected patients, hospitals and healthcare providers have been inundated with cases. Given the major role front-line healthcare professionals (HCPs) play in combating the epidemic, it remains critical to emphasize the need for appropriate personal protective equipment (PPE) and well-designed protocols to decrease the risks of transmission and infection.

Evidence has demonstrated that SARS-CoV-2 binds to angiotensin-converting enzyme-II receptors on the host and shares 79.6% sequence identity to SARS-CoV, the virus responsible for the SARS pandemic in 2002 (3). This virus causes an acute respiratory and gastrointestinal syndrome with human-to-human transmission through direct contact, fomites, and saliva droplets (particles >5.0 μm in diameter that travel short distances, namely 1- 2 meters, before settlings on surfaces, where viruses may remain infectious for days). Additionally, SARS-CoV-2 may be transmitted via aerosol (particles <5.0 μm that can linger in the air for longer and travel further) produced by coughing. While these are thought to be the most common modes of transmission, the virus may have other transmission routes and has been isolated in urine and stool as well as the oropharynx (4,5). Once an individual has contact with a source of transmission and is infected, the average incubation period is 4 days but may last up to 14 days until symptom onset (6).

Despite this evidence, there remains a paucity of data regarding SARS-CoV-2 and the current pandemic. A recent study by Guan et al of 1,099 cases with laboratory-confirmed infection revealed the most common manifestations of COVID-19 to be fever (88.7%), cough (67.8%), fatigue (38.1%), and productive cough (33.7%). Despite these systematic and respiratory complaints, gastrointestinal symptoms have also been noted along with other severe complications. Importantly, this study also demonstrated a mortality and infection rates of 1.4% and 3.5%, respectively, among healthcare workers (6).

Given the major rapid onset and to contain the spread of COVID-19 in São Paulo, the most populous city in South America, in March 2020, the government declared a state of quarantine. At this time, one of the buildings in the largest hospital in South America, Hospital das Clínicas da Faculdade de Medicina da Universidade de São Paulo (HCFMUSP)—part of the Brazilian National Public Health System (SUS)—was transformed into the largest treatment center for patients infected with SARS-CoV-2 worldwide. This 900-bed hospital, including a 200-bed-specific intensive care unit, was exclusively designed for patients with moderate and severe COVID-19.

Data from the middle of April demonstrated over 58,000 laboratory-confirmed cases of COVID-19 within Brazil and more than 4,000 deaths - of which 33% of cases and 41% of deaths occurred in São Paulo (7). Like many other HCP, gastroenterologists and endoscopists have risk exposure, necessitating well-designed protocols to minimize the risks of transmission. Recommendations and institutional guidelines for infection control should be established to support endoscopists. This study describes a routine protocol from a quaternary university referral center exclusively for COVID-19 patients to minimize the risk to HCP without compromising patient care.

### Healthcare System before COVID-19

Brazil has approximately 211 million inhabitants and provides healthcare in a two-tier system. The SUS is Brazil's publicly funded health care system and is entirely free of any cost to any individual, including foreign nationals. The other tier of the healthcare system is based on a private insurance payer model, which accounts for approximately 25% of the population. Approximately 36% of the 46 million residents of São Paulo have private insurance, with the remaining population utilizing the public healthcare system (8-10).

The HCFMUSP public health institution complex is a quaternary-care hospital that occupies a total area of 600 thousand square meters with 2,400 beds distributed among its nine specialized institutes and two auxiliary hospitals, namely, the Central Institute, Ambulatory building, Psychiatry Institute, Orthopedics and Traumatology Institute, Physical Medicine and Rehabilitation Institute, Children’s Institute, Heart Institute, Radiology Institute, and Cancer Institute, and Suzano Auxiliary and Cotoxo Auxiliary Hospitals. The Central Institute of Hospital das Clínicas (ICHC) includes most of the HCFMUSP Complex's specialties - providing 31 clinical and surgical specialties, of which, one is the endoscopy unit.

### Hospital Structure during the COVID-19 Pandemic

**Figure 1:**
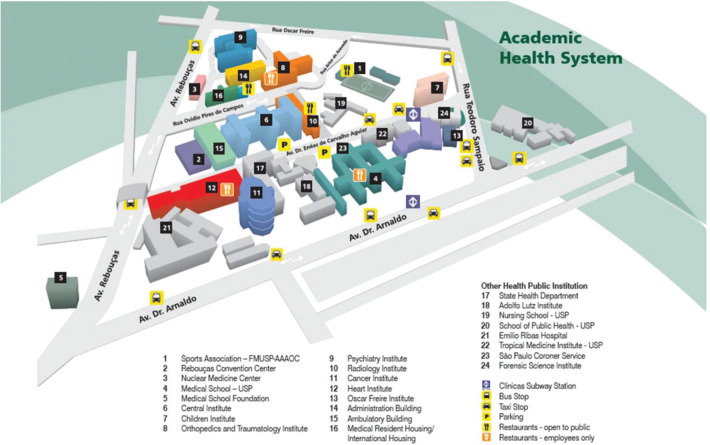
The Hospital das Clínicas da Faculdade de Medicina da Universidade de São Paulo (HCFMUSP) Complex.

On March 30, 2020, elective hospitalizations, surgeries, and procedures were suspended, and the ICHC was evacuated to become a specialized COVID-19 hospital. All patients were transferred to other buildings of the complex (non-COVID-19) (Figure 1).

Urgent endoscopic examinations and procedures for non-COVID-19 patients continue to be performed in the endoscopy unit. However, patients with laboratory-confirmed COVID-19 or those with high suspicion for the virus infection are now admitted to the ICHC - a dedicated block of the Surgical Center of this building. Isolating these patients from the standard endoscopy unit aims to reduce viral transmission and prevent spread among hospitalized patients. Each unit and team carry a specific responsibility and specific team as described below.

All HCP were vaccinated against influenza. Fellows of all specialties were assigned to exclusive clinical care for COVID-19 patients. Employees from all sectors were divided between the institutes and have remained redeployed to those sectors until such a time that the pandemic has become better controlled. The front-line staff in the isolation area underwent specific training to ensure the adequate PPE use and distance learning was provided for all employees (11).

### Recommendations for Infection Prevention and Control during Endoscopic Procedures

Recommendations have been published by the Brazilian Society of Gastrointestinal Endoscopy (12), the Brazilian Health Regulatory Agency (Anvisa) (13), the Interamerican Society of Digestive Endoscopy (14), the American Society for Gastrointestinal Endoscopy (15), The World Endoscopy Organization (16), the European Society of Gastrointestinal Endoscopy (17), the European Society of Gastroenterology and Endoscopy Nurses and Associates (17), and WHO (18-21). Among these varied regulatory societies and organizations, it is important to emphasize and summarize the following:

1. The need to establish clinical triage to evaluate all patients upon admission. Early identification of potential COVID-19 patients should be instituted to allow isolation of potentially infected individuals from other patients. Screening questions and clinical triage should include assessment for symptoms of infection (i.e., fever, cough, dyspnea, fatigue, myalgia, sputum production) as well as known exposures or recent travel.
2. Standard precautions, including appropriate hand and respiratory hygiene, PPE use, appropriate waste management, environmental cleaning, and patient-care equipment sterilization procedures, should always be followed.
3. A significant reduction (if not total suspension) in elective examinations should be implemented to reduce the circulation of people and thus transmission.
4. Adopt measures for schedule spacing (i.e., social distancing) to avoid crowding and areas containing more than six individuals. It is necessary to maintain a minimum distance of 1 meter between patients in waiting rooms and to provide material for hand hygiene.
5. All individuals should be considered suspected or at a risk of COVID-19 (i.e., at high-risk of community transmission). Patients, family members, and HCP should use contact and droplet precautions before entering the patient room. Visitors should be avoided.
6. The correct use of PPE is the most important method to avoid HCP contamination.
7. If patient transportation is required, we recommend the use of predetermined routes to reduce exposure to HCP, other patients, and visitors. Logistical requirements and planning should be rapidly put in place and ensure that patients and transport personnel wear medical masks at all times.
8. Aerosol-generating procedures have been associated with an increased risk of COVID-19 transmission; thus, special caution must be taken and N95/PFF2 masks are recommended.
9. Although the risk has not yet been clearly defined, due to the high potential for aerosol generation in digestive endoscopy procedures, we recommend that all digestive endoscopy procedures—esophagogastroduodenoscopy, colonoscopy, enteroscopy, endoscopic retrograde cholangiopancreatography (ERCP), and endoscopic ultrasound (EUS)—be considered aerosol-generating procedures (AGPs).
10. AGPs should be preferably performed in an adequately ventilated room (i.e., natural airflow ventilation of at least 160 L/s per patient or in negative-pressure rooms with at least 12 air changes per hour and controlled direction of airflow when using mechanical ventilation).
11. Only essential staff should be present during endoscopy procedures.
12. After any procedure, frequent cleaning and disinfection of the examination room surfaces are indicated. After cleaning, no waiting time is required to reuse the room.
13. Terminal cleaning must be performed at the end of the day and between procedures.

### Outpatient Management and Examination of Hospitalized Patients in Non-COVID Institutes

The temporary suspension of elective examinations and maintenance of services only in cases of non-postponable elective procedures (i.e., stricture dilation, cancer staging or treatment, dysphagia, enteral feeding tube placement, and obstructive jaundice without cholangitis) and urgent/emergency examinations (i.e., acute gastrointestinal bleeding, ingestion of caustic substances, foreign body removal, and acute cholangitis) can reduce the use of PPE in elective patients and the circulation of individuals, which may consequently impact virus transmission (22,23). All patients must be contacted by fellow doctors via telephone before the procedure and instructed to reschedule the examination if they have symptoms of respiratory infection or if the examination has an elective nature. If the procedure cannot be postponed, it is critically important to adopt measures for schedule spacing (i.e., social distancing) to avoid crowding. Patients should also be informed to arrive with as few companions as possible to reduce risks to both patient and HCP. This social distancing policy should also apply to the waiting room. We advise adjusting the seating in the waiting room to ensure a minimum distance of 1 meter between seats if possible. It also remains imperative to provide material for hand cleaning (i.e., alcohol-based solutions or soap and water) and clear written information regarding cough and general hygiene etiquette. The frequency of disinfectant treatment of public surfaces should also be increased (21,24). In areas of community spread, all outpatients should be tested for COVID-19 before endoscopic procedures or should be treated as though they are positive. Full or enhanced PPE should be used for all cases.

### Examinations on Patients Admitted to ICHC (COVID-19-positive Patients)

**Figure 2:**
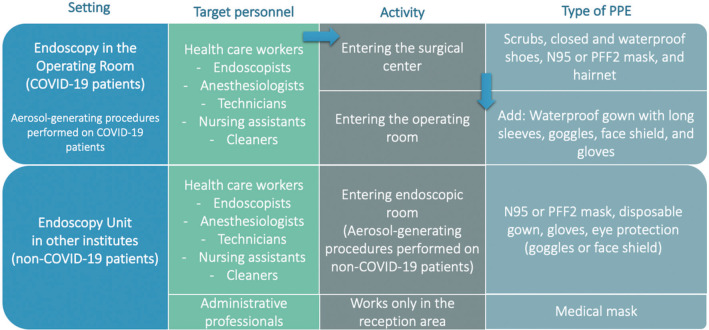
PPE Recommendations. Adapted from the US Centers for Disease Control and Prevention (CDC) and World Health Organization (WHO) (20,25). Abbreviations: PPE, personal protective equipment; COVID-19, coronavirus disease 2019.

As all patients admitted to ICHC have a high clinical suspicion for COVID-19 or laboratory-confirmed disease with moderate or severe symptoms, examinations should be performed exclusively in a room dedicated to endoscopic procedures. PPE recommendations for each employee are specified in Figure 2 (20,25).

### Supervised Usage of PPE

It is critical to ensure team members and all HCP perform appropriate hand cleaning and proper PPE donning and doffing. In the designated &quot;donning/doffing room,” PPE removal is assisted by a trained nurse. Gowning down has the greatest risk of contamination. Therefore, we emphasize repeated preparation and supervision by the nursing team or a colleague and suggest that individuals be signed off on the process by a supervisor before performing any examinations or evaluations of suspected patients. The doffing procedure is conducted under the guidance and supervision of a qualified observer who uses verbal commands to confirm each step of doffing PPE, assisting when necessary, and visually confirming the completion of each step. In institutions without a dedicated &quot;donning/doffing room”, we recommend paired PPE removal, in which endoscopists and assistants alternate as &quot;removers&quot; and &quot;checkers&quot;, always following the safety and standardized steps. With this regimen, there have been no cases of contamination in our endoscopy staff. We believe that the implementation of routine protocols such as described here can lead to decreased virus transmission and ensure adequate and appropriate use of PPE.

### Recommended Sequence for PPE Use

Based on CDC and WHO recommendations, more than one method may be acceptable. We suggest the following precautions for PPE placement and removal.

### Appropriate PPE Application (Donning) of PPE (Figure 3):

1. Perform hand hygiene using hand sanitizer with an alcohol-based solution or soap and water.
2. Dress in scrubs. Ensure the size is correct.
3. Wear close-toed and waterproof shoes.
4. Perform hand hygiene gain using hand sanitizer or soap and water.
5. Don filtering N95 or PFF2 facepiece respirators. Place the mask over the nose and mouth. Mold the nosepiece. Adjust the top strap to fit comfortably above the ears. Adjust the bottom strap to fit comfortably below the ears. If you feel air blowing at the edge of the mask, readjust the mask. Perform hand hygiene after touching the mask.
6. Place a hairnet over the top of the head.
7. Apply goggles to cover the eyes and brow.
8. Put on the face shield.
9. Don a disposable long-sleeved waterproof gown. Tie all ties with the help of an assistant.
10. Put on gloves. Gloves should cover the wrist.
11. HCP can then enter patient room.

### Appropriate PPE Removal (Doffing) (Figure 4):

1. Remove gloves. Ensure that glove removal does not cause additional contamination of the hands. Dispose of gloves in infected waste.
2. Remove gown. Untie all ties. Reach up to the shoulders and carefully pull gown down and away from the body. Dispose gown of as infected waste.
3. HCP can then exit the patient room.
4. Perform hand hygiene.
5. Carefully remove the face shield and goggles by grabbing the strap and pulling upwards and away from the head. Do not touch the front of the face shield or goggles.
6. Remove hairnet. Dispose of the hairnet as infected waste.
7. Remove the bottom strap of the respirator by touching only the strap and bring it carefully over the head. Grasp the top strap and bring it carefully over the head and then pull the respirator away from the face without touching the front of the respirator. Store it in a breathable paper-like packaging or dispose of it properly.
8. Perform hand hygiene.
9. Remove scrubs and waterproof shoes.
10. Perform hand hygiene.
11. HCP can then exit the hospital.

### Endoscopy Teams (COVID and non-COVID)

The endoscopy staff (physicians, fellows, nurses, technicians, and staff) is divided into “COVID” and “non-COVID” teams. The former exclusively serves patients admitted to the ICHC and the latter serves outpatients in the endoscopy unit. Each team consists of a physician assistant, one nursing technician, and one fellow. The scale is divided into 24-hour shifts. HCP on the COVID team are not assigned to more than 24 hours/week, with an interval of at least 1 week between shifts from COVID to non-COVID teams. These administrative measures were designed to prevent long exposure times and cross-infection.

Endoscopy teams working in the ICHC (COVID building) should not have personal contact during the pandemic period with different scales or on duty to reduce the risk of cross-contamination and decrease the likelihood of transmission. Teams must contain as few HCP as possible to limit exposure. Workstations such as the report room, computers, and folders, must be sanitized at the beginning of shifts, between individual uses, and at shift conclusion. All endoscopist faculty and staff over 60 years of age were temporarily redeployed to only teaching and scientific activities and did not provide direct care to COVID-19 patients.

### Endoscopy in the Operating Room: Patients with COVID-19

**Figure 5:**
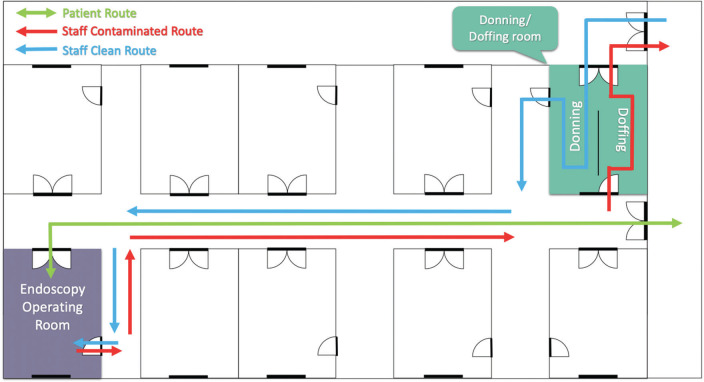
Organization of the Operating Room Block for Coronavirus Disease 2019 (COVID-19) Patients. There is an exclusive operating room for endoscopic procedures. Entering and leaving the block requires passing through the dressing room. A single patient can move down the corridor at a time. According to Hospital das Clínicas da Faculdade de Medicina da Universidade de São Paulo (HCFMUSP) recommendations.

The COVID-19 endoscopy team is scheduled based on an on-call system. When notified, these providers arrive directly at the operating room. Importantly, this team is prohibited from moving through non-COVID-positive areas of the hospital to minimize transmission. When the team arrives at the surgery center, HCP must go through the locker room, then into the donning/doffing room to properly don PPE, and finally to the patient's room (Figure 5). This specific operating room is reserved for endoscopic examinations and procedures on COVID-19 patients. Ideally, this room should be a negative pressure room and be equipped with an endoscopy trolley, fluoroscopy, patient stretcher, and system to deliver anesthesia. Although this is our recommendation, we do not have a currently negative pressure room for this process in our hospital.

**Figure 6:**
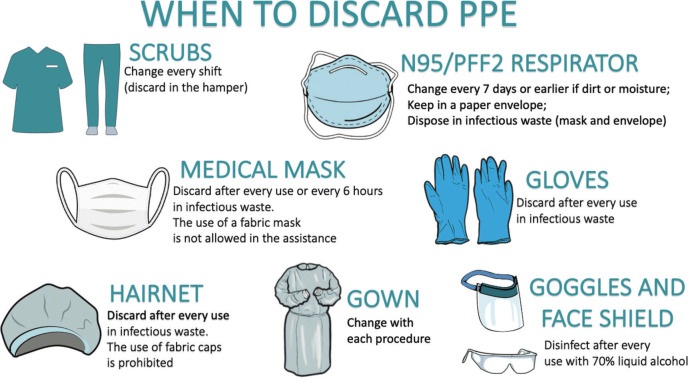
PPE Disposal Suggestion. According to Hospital das Clínicas da Faculdade de Medicina da Universidade de São Paulo (HCFMUSP) recommendations. Abbreviations: PPE, personal protective equipment.

The room must be set up, and the endoscope must be tested before patient arrival. We recommend that only one patient be transported into the aisle of the operating room at a time. All HCP involved in patient transport must adopt standard precaution measures and the patient must wear a surgical mask throughout the course to the operating room. The anesthesiology team is responsible for sedation or general anesthesia. Topical spray anesthetics to numb the throat should be used in favor of a lidocaine swallow (26). During anesthetic induction, only the anesthesia team should remain in the room with non-essential providers absent to limit transmission. Once induction and general anesthesia are performed, the other providers can then enter the room to begin the procedure. After the procedure, the endoscopist and other non-essential providers should exit the room before extubation by the anesthesia provider. The durability and proper disposal of PPE are shown in Figure 6.

### Endoscopic Procedures in the Endoscopy Unit: Non-COVID-19 Patients

The endoscopy unit has been reorganized. There has been a redistribution of all employees in the sector (administrative, nursing, medical, and cleaning) to the absolute minimum required for patient care and support. The number of examination rooms has also been reduced from eight to three; one for esophagogastroduodenoscopy, one for colonoscopy, and the other for ERCP or EUS and other advanced therapies.

### Care during Endoscopic Examinations

The use of procedural oxygen masks (oxygen mask with specific openings for endoscope access) should be considered for all upper endoscopy procedures to decrease aerosolization during these procedures. Intubation should also be considered for longer cases to limit ongoing aerosolization (26).

Patient fluids may spray or aerosolize during the examination, especially when manipulating the suction/insufflation valves and when removing accessories from the endoscope's working channel. Single-use biopsy valves should preferably be utilized when possible. This step of the procedure has a high risk for contamination. To properly remove accessories, we recommend the use of the “double gauze technique,” where the endoscopist holds a gauze next to the working channel and the assistant removes the accessory by cleaning the entire length of the accessory with another gauze, keeping a short distance from the endoscopist's hand. Extra care should be taken at the end to avoid the “whip” effect of the accessory to prevent spillage of fluid or droplets.

At the end of the examination, the endoscopist must perform precleaning. Enzymatic detergent cleaning solutions should be suctioned for 10-15 seconds or until the suction tube returns clean or non-turbid to avoid leakage of mucus and blood. We recommend also wiping gauze along the outer surface. Next, the endoscopist should place the endoscope in an appropriate tray identified as contaminated. Next, a technician wearing clean gloves and appropriately donned PPE should disconnect the endoscope from the processor, turn off the buttons of the processor, place the instrument in a sealed box, and send it to the appropriate area for high-level disinfection (12).

### Reprocessing of Gastrointestinal Endoscopes

There are no specific recommendations for the decontamination of devices during the SARS-CoV-2 outbreak. The recommendations are the same as those for the high-level disinfection of endoscopy devices. Training should be reinforced and meetings held with employees, emphasizing the importance of strictly following the endoscope's reprocessing policy as a safe and efficient method to prevent the spread of viral infection.

### Cleansing Endoscopy Rooms

The cleaning of the procedure room must follow the protocols established by the respective institutions. The room must be closed for a set time following the procedure that depends on the use of negative pressure and the air exchange rate. SARS-CoV-2 behavior and risk of transmission on inanimate surfaces are not yet fully understood. Surfaces such as the endoscopy trolley, processor, worktable, and floor must be cleaned systematically and periodically to reduce the risk of infection. UV light may also be used to cleanroom surfaces and air. The stretcher must also be sanitized after each examination.

## CONCLUSION

The present COVID-19 pandemic has ushered in an unprecedented era of quality control, necessitating advanced safety precautions and the need to ensure the adequate protection of HCPs. Like many other medical specialties, the field of digestive endoscopy has had to reevaluate the safety practices at each institution to ensure the safety of professional healthcare staff, patients, and, consequently, society. Our established COVID-19-specific administrative and routine protocols can be replicated or adapted to multiple institutions and endoscopy units worldwide. Based on these recommendations and summary protocol, we aim to improve patient and HCP safety.

## AUTHOR CONTRIBUTIONS

Franzini TAP, Kotinda APST were responsible for the data acquisition and interpretation, manuscript drafting, review, and final approval. Moura DTH was responsible for the data analysis and interpretation, and manuscript review. Badana MLV, Medeiros MS, Lima PGR, Mello BF, Kayano RP, Carmona MJC, Rocha MC and Campos AF were responsible for the development of the assistance protocol for COVID-19 patients at HCFMUSP surgical center. McCarty TR, Guimarães T, Moura MLN and Thompson CC were responsible for the manuscript drafting, review, and final approval. Moura EGH was responsible for the data analysis, and manuscript interpretation, drafting, review and final approval.

## Figures and Tables

**Figure 3:**
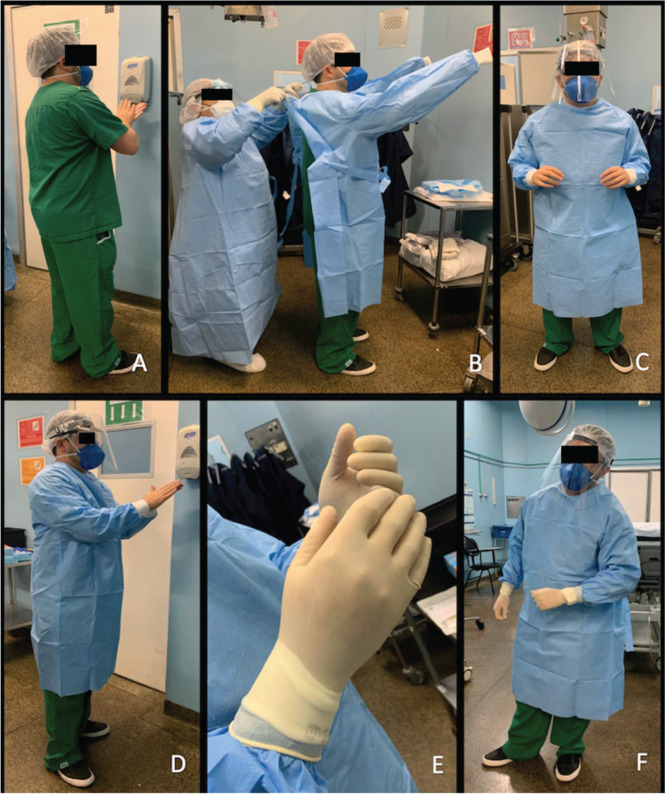
Guidance for Donning PPE to be used by Healthcare Professionals during Endoscopic Procedures for Patients with Confirmed/Under Investigation for COVID-19. First, the healthcare professional dons scrubs, closed and waterproof shoes, N95 or PPF 2 mask, and hairnet in the locker room. Only then should the healthcare professional enter the surgical center. Before entering the operating room, the healthcare professional must wear PPE in the dressing room (donning/doffing room): A. hand hygiene; B. don the long-sleeved waterproof isolation gown; C. put on goggles and face shield; D. hand hygiene; and E. put on gloves that fully cover provider's wrist. F. The healthcare professional may now enter the operating room. Abbreviations: PPE, personal protective equipment; COVID-19, coronavirus disease 2019.

**Figure 4:**
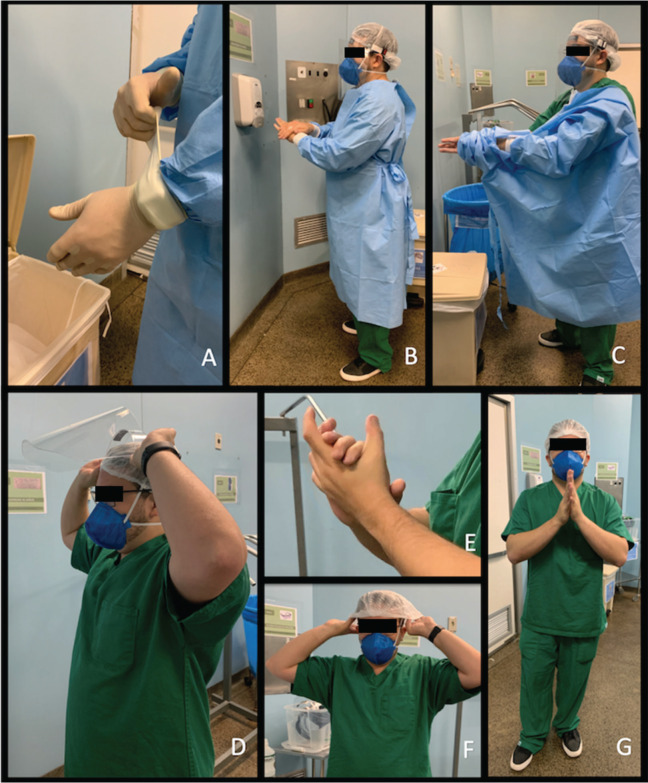
Guidance on Removing PPE to be used by Healthcare Professionals during Endoscopic Procedures for Patients with Confirmed/Under Investigation for COVID-19. After the procedure, the professional should go to the dressing room (donning/doffing room) and: A. remove gloves; B. hand hygiene; C. remove and discard gown; D. remove face shield and googles; E. hand hygiene; F. discard hairnet and put on a new one; and G. hand hygiene. After these steps, the healthcare professional may leave the surgical center and enter the locker room. Abbreviations: PPE, personal protective equipment; COVID-19, coronavirus disease 2019.
